# Chemical Assistance in Refolding of Bacterial Inclusion Bodies

**DOI:** 10.1155/2011/631607

**Published:** 2011-08-01

**Authors:** Mona Alibolandi, Hasan Mirzahoseini

**Affiliations:** Medical Biotechnology Research Center, Pasteur Institute of Iran, Tehran 13169 43551, Iran

## Abstract

*Escherichia coli* is one of the most widely used hosts for the production of recombinant proteins but insoluble expression of heterologous proteins is a major bottleneck in production of recombinant proteins in *E. coli*. *In vitro* refolding of inclusion body into proteins with native conformations is a solution for this problem but there is a need for optimization of condition for each protein specifically. Several approaches have been described for in vitro refolding; most of them involve the use of additives for assisting correct folding. Cosolutes play a major role in refolding process and can be classified according to their function as aggregation suppressors and folding enhancers. This paper presents a review of additives that are used in refolding process of insoluble recombinant proteins in small scale and industrial processes.

## 1. Introduction

The expression of recombinant products is an essential method for producing target proteins and one of the most important unit processes for production of therapeutic protein and structural study [1–6]. There are different expression systems for producing recombinant protein in biotechnology industry. Expression in mammalian cells produces active recombinant proteins that have posttranslation modification but expression by this system is time consuming and expensive. Although bacteria still represent a convenient production system, most of recombinant polypeptides produced in prokaryotic hosts undergo misfolding or incomplete folding processes that usually result in their accumulation as insoluble aggregates, which called inclusion bodies [7–9]. All these studies of recombinant protein expression in *Escherichia coli* showed that inclusion body formation is the rule rather than the exception.

*In vitro* refolding is required for obtaining active protein from inclusion body aggregates which consist of four main steps: inclusion body isolation, solubilization, refolding, and purification. Because of high density of IBs, they separated easily by centrifuge. After isolation of the IBs, the proteins contained in the inclusion bodies need to be solubilized, usually by high concentration of denaturing agents such as urea or guanidinium chloride (Gn-HCl). Then, solubilized IBs are subjected for refolding in specified conditions. Refolding of proteins from inclusion bodies is influenced by different factors including solubilization method, removal of the denaturant, and assistance of refolding by co-solute or additives. The refolding condition is critical in order to obtain acceptable amounts of active protein. It has been known that additives, especially low molecular weight compounds, may significantly enhance the yield of the refolding process. In many cases, inclusion body protein can only be successfully refolded by making use of these effects.

## 2. Cosolute Assistance

Low molecular weight additives are usually added to refolding buffer to promote the refolding process. In the case of refolding by dilution, there is a residual concentration of Guanidine HCl or urea in refolding buffer. This low concentration of denaturants holds aggregate prone structures of target protein in soluble and flexible state; thus refolding yield efficiency improves. Due to this reason, additional to residual concentration of denaturants, additives should be added to refolding buffer to decrease the degree of aggregates and misfolds. Co-solute may be classified into two groups: folding enhancer and aggregation suppressor. In principle, folding enhancer enhances protein-protein interaction, while aggregation suppressor reduces side chain interactions [4, 17].

The folding enhancers facilitate interaction between proteins and thus increase the stability of proteins whereas aggregation suppressor reduces side chain interaction of folding intermediates without interfering with refolding process [4]. Common additives used in refolding of recombinant proteins are shown in Table 1.

## 3. Arginine (Arg)

Arginine is one of the useful reagents that are effective in assisting refolding of recombinant proteins from inclusion bodies. It is an aggregation suppressor that improves refolding yield; however, the mechanism of arginine action during refolding is still unclear. ARGININE was first used in refolding of human tissue type plasminogen activator [18, 19]. Since then, it has been used for refolding of a variety of proteins including casein kinase II, [20], Fab antibody fragments [18, 21], growth hormone [22], human gamma interferon, [23], human matrix metalloproteinase-7 human neurotrophins [24] human p53 tumor suppressor protein [25], interleukin-21 [26], interleukin-6 receptor [27], lysozyme [28], single-chain Fv fragments [29], and single-chain immunotoxins [18, 30]. However, arginine may act as a protein-denaturant, which limits the expansion of its applications. Yancey et al. [31] showed that the activity and stability of certain enzymes were perturbed by arginine and concluded that arginine is a protein-destabilizer. Xia et al. [32] have made a similar observation and showed that fluorescence properties of aminoacylase are perturbed by arginine. They interpreted the observed effects of arginine in terms of denaturing property of the guanidinium group, which makes guanidine hydrochloride (Gn-HCl) a strong denaturant. But arginine differs from Gn-HCl in the mode of interactions with proteins.

## 4. Cyclodextrins (CDs)

Cyclodextrins have been reported to suppress aggregate formation during the refolding of recombinant proteins. These cyclic glucose macromolecules have hydrophobic cavities and functional groups that can interact with polypeptide chains by weak forces similar to those involved in protein folding. CDs may interact with proteins via hydrophobic forces, hydrogen bonding, and Van der Waals interactions. Charged chemically modified CDs can also bind to the folding polypeptide chain via electrostatic interactions. CDs have been investigated in both dilution additive and artificial chaperone-assisted modes. CDs have been used as stripping agents for removal of detergents in proteins refolded with surfactants [33, 34]. CDs have been used for refolding of recombinant-human growth hormone [35] and *α* amylase [36].

## 5. Polyethylene Glycol (PEG)

Polyethylene glycol is one of the water-soluble polymers that are used for refolding of recombinant proteins [37]. PEG, a polyhydric alcohol, like other polyols is known to stabilize protein conformations as well as to increase the rate of *in vitro* protein refolding [38, 39].

 It enhances the recovery of active protein by preventing aggregation during refolding [40]. PEG improves *in vitro *structure formation, which can block aggregation by combining with a molten globule folding intermediate. It stabilizes proteins by chemical modification [41, 42]. PEG binds an intermediate in the folding pathway of carbonic anhydrase, therefore prevents self-association and promotes correct refolding [43–45]. PEG interacts with the hydrophobic side chains exposed upon unfolding at high temperatures [46]. PEG has been successfully used for refolding of interferon [47], xylanase [48], and insulin-like growth factor (IGF-1) [49] from inclusion bodies.

## 6. Proline (Pro)

The role of proline was studied for the first time in *in vitro* refolding of bovine carbonic anhydrase. Proline inhibited bovine carbonic anhydrase aggregation and enabled the protein to return to its native structure. It was proposed that proline acts as a protein-folding chaperone due to the formation of an ordered, amphipathic supramolecular assembly [50]. Later, proline was found to prevent aggregation during hen egg-white lysozyme refolding. Also proline has been used during refolding of arginine kinase [32] and aminoacylase [51]. Proline inhibits protein aggregation by binding to folding intermediates. Proline (1 M) was also found to act as a creatin kinase (CK) folding aid [52]. Results further indicate that proline possibly binds to and stabilizes folding intermediates and reduces the hydrophobic surface of CK, thus inhibiting protein aggregation and improving the final yield of CK. The unusual properties of proline may be due to hydrophobic stacking in aqueous solution and intermolecular self-association [53]. The hydrophobic nature of proline covers hydrophobic region of the proteins, through which this amino acid effectively suppresses protein aggregation [54].

## 7. Ammonium Sulfate ((NH_4_)_2_SO_4_)

Salts can stabilize proteins through nonspecific electrostatic interactions at low concentration, dependent on the ionic strength of the medium [55]. At high concentrations, salts exert specific effects on proteins depending on the type and concentration of the salts, resulting in either the stabilization or destabilization of proteins, or even denaturation [56]. Salts usually increase interfacial tension between the protein surface and solvent [57], which leads to increase the solubility of proteins and aggregates, although the stabilizing effect of salts on protein structure is closely correlated to the salting-out effect of salts. 

Ammonium sulfate is widely used as a precipitant for protein purification and for crystallization, as this salt can prevent aggregation. Ammonium salts have been reported to prevent heat-induced aggregation of lysozyme [58]. Also magnesium chloride is another example of salts that has been used in refolding of recombinant proteins [59].

## 8. Polyols

Polyols vary in length of carbon chain and number of hydroxyl groups. Polyols used as additives for enhancing refolding yield and stabilize protein structure. The structure-stabilizing polyols are supposed to lead to compact folded state. Bolen and Baskakov demonstrated that the addition of certain osmolytes results in the compaction of RNase A [60]. Polyols have been shown to stabilize the acid-unfolded molten globule state of cytochrome *c* by enhancing hydrophobic interactions that overcome electrostatic repulsion at low pH between charged residues [61].

Glycerol has long been known as a protein stabilizer, and its stabilization mechanism has been shown by Timasheff [62]. Glycerol leads to the enhancement of hydrophobic interactions as a consequence of an increase in the solvent ordering around proteins. Comparing with the other polyols, glycerol is a mild stabilizer of protein conformation. Unlike other polyols, Glycerol has unusual properties that decreases the surface tension of water but increases its viscosity [63, 64]. Increasing glycerol concentrations are known to increase the stability of proteins at all concentrations [62, 65] although it decreases refolding at high concentrations due to its ability to considerably slow down the kinetics of refolding, which could result in off-pathway aggregation. An optimum glycerol concentration seems to be critical for striking a balance between its stabilizing effect governed by thermodynamic principles and its effect on the kinetics of folding glycerol successfully used in refolding of lysozyme [66].

Sorbitol is another polyol that has been shown to reduce aggregation of nucleocapsid protein of rhabdovirus after its expression in *Escherichia coli *[67]. Sorbitol is likely to exert its effect on folding by altering the structure and properties of water around the folding protein molecule. 

Erythritol is also a protein structure stabilizer and studies showed that it stabilizes proteins to lesser extents compared with sorbitol [64]. The study on citrate synthase aggregation kinetics during refolding in erythritol and other polyols showed the ability of the polyols to stabilize proteins depending on the number of hydroxyl groups in them [68].

## 9. Stimuli-Responsive Polymers

Recently it has been shown that smart polymers can act as a pseudochaperonins and precipitation of the protein from its denatured solution by such polymers can yield refolded active protein [69, 70]. Such polymers exist in soluble state in aqueous buffers and can be made insoluble by application of a stimulus. The latter can be a pH change, temperature change, or a change in the concentration of chemical species. This process is reversible.  Table 2 represents the smart polymers which are used in refolding of recombinant proteins.

## 10. Conclusion

Obtaining a good amount of active protein at low cost is the main objective in *in vitro* refolding of recombinant protein from bacterial inclusion bodies but there is no single refolding technique or method that satisfies all protein refolding processes. With providing a good condition for *in vitro* refolding of inclusion body, bacterial system could be an excellent alternative to the mammalian expression system or other expression systems that can directly generate active proteins with a complex disulfide bond structure. In this process, the solubilized target protein is subjected to refolding using different methods. Increasing protein concentrations during the refolding process improves protein refolding and the final refolding yield. Refolding aiding chemicals are helpful agents for increasing protein concentration especially in dilution method. Therefore, application and optimization of additives concentration is a main step in protein refolding and should be done for each protein individually.

## Figures and Tables

**Table 1:** Examples of buffer additives which may be used to facilitate protein refolding (see text references).

Additive	Concentration	Effect
CHAPS	30 mM	Detergent
EDTA	20 mM	Chelate
Glycerol	10–50%	Stabilizer
Guanidine HCl	0.1–1 M	Chaotroph
L-arginine	0.4–0.5 M	Stabilizer
Laroylsarcosin	Up to 4 M	Detergent
MgCl_2_/CaCl_2_	2–10 mM	Cation
NaCl/Ammonium sulfate	0.2–0.5 M	Salt
Non-detergent sulfo betain	Up to 1 M	Solublizer
PEG 3350	Up to 0.5% W/V	Osmolyte
SDS	0.1%	Detergent
Sodium citrate/sulfate	0.2–0.5 M	Salt
Sucrose/glucose	Up to 0.75 M	Stabilizer
TMAO	1–3 M	Osmolyte
Tris	0.4–1 M	Buffer
Triton X-100	0.1–1%	Detergent
Tween-80	0.01%	Detergent
Glycine	Up to 1 M	Osmolyte
Proline	Up to 1 M	Osmolyte
Urea	0.1–2 M	Chaotroph

**Table 2:** Lists of smart polymers which are used in refolding procequres.

Smart polymer	Sensitive	Protein	Reference
Eudragit S100	pH	hbFGF/haFGF	[10]
Alginate	Ca^++^	Lipase Amilas	[11, 12]
Poly(propylene oxide)−phenyl−poly(ethylene glycol)/PPO (*n*) 33, PPO (*n*) 50	Temperature	Carbonic anhydrase	[13, 14]
Poly(*N*-isopropylacrylamide)	Temperature	Carbonic anhydrase lysozyme	[15, 16]
